# Expert opinion: Seeking truth through inquiry: A discussion on the online news from *Science* on November 21, “Exclusive: CDC to end all monkey research”

**DOI:** 10.1002/ame2.70132

**Published:** 2026-01-14

**Authors:** Zhiguang Xiang, Chuan Qin

**Affiliations:** ^1^ Chinese Academy of Medical Sciences and Peking Union Medical College Beijing China; ^2^ Chinese Association for Laboratory Animal Sciences Beijing China

When it comes to journalism, in today's highly informationalized era, it is not unjustified for news reports to employ exaggerated or emotionally charged language to capture audience attention, as it is also natural for journalists to infuse their personal sentiments into their news articles.^1^

Given the strong recognition among researchers, of the academic influence wielded by top‐tier journals like *Science* and *Nature*, we pay special heed to information disseminated through *Science*'s online news. On November 21, 2025, *Science* published an online news report titled “Exclusive: CDC to end all monkey research,” with a subtitle stating, “Studies related to HIV and other infectious diseases will be phased out, sources say; fate of the agency's animals remains unclear,” authored by David Grimm, an online news editor at *Science* magazine.^1^

Grimm learned that the US Centers for Disease Control and Prevention (CDC) may have received instructions, potentially from government authorities, to phase out all of their monkey research by the end of this year. In his report, Grimm described its correlation with the policies of the new US administration, suggesting that the directive may stem more from political commitments made by the government. Early in the year, agencies such as the FDA also released similar information regarding the reduction of animal use and the promotion of organoids and tissue chip applications.^2^ Despite searching the CDC's official website, we found no information pertaining to this directive. *Science* magazine only received an email response from the CDC stating, “CDC regularly evaluates its research programs, including non‐human primate research, and strives to use non‐animal research methods when feasible.” Grimm's report also cited opinions from animal rights activists and life science scholars on this matter, revealing divergent attitudes. While we do not doubt the authenticity of the news, we find it objectionable that some disseminators deliberately expand its scope with ambiguous headlines, creating misunderstandings. There is an urgent need for more professionals to promptly clarify and correct such misconceptions.

The United States is home to numerous institutions conducting scientific research using nonhuman primates, including not only the CDC but also several nonhuman primate research centers supported by the NIH. Any research involving laboratory animals must undergo rigorous review by the Institutional Animal Care and Use Committee (IACUC). The initiation of animal experiments is far from a hasty decision; similarly, the cessation of ongoing animal research should not be arbitrarily prompted. This raises a crucial question: What impact can government or departmental directives exert within established research governance and review processes? Our stance is that research should always be guided by scientific evidence and ethical norms, rather than being supplanted by administrative orders that bypass professional evaluations and compliance procedures.

The reduction of laboratory animal use has been a consensus among life science researchers since the introduction of the 3R principles last century. In China, we have established a standardized system for laboratory animals, ensuring animal welfare in terms of environment, nutrition, animal quality and health, and animal experiment review. With the advancement of new technologies, the trend toward reducing laboratory animal use or employing alternative technologies in experiments that can be conducted without live animals is inevitable. For instance, in vitro human skin models can replace rabbit skin tests in cosmetic research,^3^ human liver tissue chips can identify 87% of hepatotoxic drugs,^4^ and big data‐based artificial intelligence tools hold promise in predicting drug efficacy and toxicity.^5^

However, life science research has not yet developed technologies that can entirely replace animal experiments. This implies that long‐term, systematic comparative medical investigations on “laboratory animals” remain unavoidable. Consider a practical scenario: if a novel drug undergoes efficacy and safety testing solely on cells or organoids composed of multiple cell types, without preclinical testing on animals, particularly nonhuman primates closely related to humans, how can this drug proceed to clinical trials? Who can serve as the initial human subjects? Who would bear the risks associated with being the “first trial participants”? This poses a new ethical dilemma: When the evidence chain primarily derives from in vitro or computational models, how can risks be identified, quantified, and allocated? How can informed consent and subject protection be realized?

We must also acknowledge the limitations of animal models. Regulatory agencies, such as the U.S. FDA, have repeatedly cautioned that drugs effective in preclinical animal experiments may not necessarily prove effective in clinical trials; furthermore, drugs deemed “safe” in preclinical studies may still exhibit toxic reactions in humans.^2^ However, if even the preclinical animal research is removed, risk assessment will rely more heavily on uncertain extrapolations and assumptions. After all, “Shennong tasting a hundred herbs” is merely a legend; the robust development of modern medicine still hinges on crucial evidence provided by laboratory animals.

Therefore, as practitioners in laboratory animal science, we must not only prioritize the research and translation of alternative technologies and new methodologies but also avoid simply pitting them against animal experiments. As Professor Kent Lloyd argued in *Nature* magazine last month, novel alternative methods and animal experiments are not mutually exclusive but rather complementary in technological applications.^6^ The application of artificial intelligence, tissue chips, and other technologies can partially replace live animal experiments, while the development of these technologies also necessitates comparative medical research with animal experiments. Our quest to unravel the mysteries of life, particularly investigations grounded in animal models, should not cease but rather delve deeper within ethical boundaries. By enhancing humanized modifications and disease model establishment, we can improve the simulation of human disease development patterns. Simultaneously, in the digital age, practitioners in laboratory animal science must also understand the demands of digital technologies and standardize the collection of comparative medical data in our work, providing accurate comparative medical data on human diseases for mathematical models. Only in this way can we, as practitioners in laboratory animal science, eventually “smile among the flowers” when artificial intelligence truly simulates biological responses in humans or animals.

## AUTHOR CONTRIBUTIONS

**Zhiguang Xiang:** Writing—original draft. **Chuan Qin:** Conceptualization; supervision; validation; writing—review and editing.

## FUNDING INFORMATION

None.

## CONFLICT OF INTEREST STATEMENT

Chuan Qin is the Chief Editor of *AMEM* and an author of this article. To minimize bias, she was excluded from all editorial decision making related to the acceptance of this article for publication.

## ETHICS STATEMENT

None.

## Data Availability

None.
